# Case of Fatal Concussion of the Brain, with the Appearances on Dissection

**Published:** 1818-02

**Authors:** John Cunningham

**Affiliations:** Surgeon, Royal Navy


### *Case of fatal Concussion of the Brain, with the Appearances on Dissection.*

By JOHN CUNNINGHAM, Surgeon, Royal Navy.

JOHN Hesdon, ætat. 24, being, as was supposed, intoxicated, fell, at six in the evening, from the waist of His Majesty's ship Gibraltar, into the fore cock-pit, and pitched on his head and right side. He was taken up motionless and insensible, and conveyed to the sick berth, where he was carefully examined. The only sign of local external injury was over the right *os mali* and temple, where the integuments appeared slightly contused, discoloured, and swelled. The whole cranium was diligently examined, but no appearance of subjacent fracture could be detected. He remained comatose; breathed as though he were in a natural sleep; and appeared completely insensible to external impressions. His extremities were quite supple, and there was a slight oozing of blood from the nose. His eyes were turned upwards; the pupils firmly contracted, and apparently insensible to the stimulus of light. The pulse was at 80, and felt natural. *Venæsectio statim ad* ℥xl from a large orifice; but without producing any relief whatever, or inducing any change in the pulse. The breathing, however, now seemed to be more laborious, and interrupted occasionally by sighing and moaning, with a kind of *catching* in his throat, which last symptom continued till death.

Finding that bleeding was attended with no good effect, a stimulating draught, with ammonia and tincture of opium, was exhibited, in part, for the power of deglutition was nearly gone; and soon afterwards he vomited up about a pint of reddish liquor, apparently the wine he had swallowed. His pulse now fell in strength, and increased in velocity; the cheeks became somewhat flushed, and the skin very hot. — *Midnight.* Continues nearly the same, excepting that his breathing is much oppressed, and seemingly interrupted by a quantity of mucus in the throat. A few grains of the sulphate of copper were exhibited, which brought up by vomiting some of the same fluid as before, with a temporary relief to the breathing. A profuse sweat now came out over the whole body; the pulse became more frequent, and was observed to be stronger in the right than in the left arm. — *Six in the morning.* Nearly in the same comatose state as before. Has passed no urine or fæces; the heat of the skin is increased. The head shaved, and cold lotions applied. It was again carefully examined, but no indication of fracture could be perceived. Cathartic swallowed with difficulty. — *Eleven o'clock, forenoon.* Finding every thing getting worse, I made a perpendicular incision through the right temporal muscle down to the zygoma of the os mali; and another half across the os frontis down to the bone; but no fissure or fracture could be ascertained: he seemed, however, a little sensible to the knife. The wounds were closed. He had a stiffness in the lower extremities, with tumultuous motions in various other parts of the body, and some strabismus. — *One*, P. M. Pulse 150; breathing more difficult; in other respects worse. The wound was again opened, and the trephine applied to the right side of the os frontis, close to the coronal suture, and a little below the ala major of the sphænoid bone. On removing the portion of bone, no blood or fluid was found. The dura mater appeared perfectly healthy, and adhered firmly to the internal surface of the removed bone. The integuments were again brought in contact, and so retained by adhesive straps. — *Eleven o'clock at night.* Pulse at least 180; breathing very laborious; heat of skin great. A vein was opened; but before a few ounces of blood were abstracted, he grew rapidly worse, and his arm was tied up. He turned sick, raised his head almost from the pillow, and immediately expired. He never spoke, nor passed either urine or fæces from the time of the accident till his death, though cathartics were exhibited by the mouth, and stimulating glysters were thrown up the rectum.

*Post mortem appearances.* — On examining the skull and brain next morning, some turgescence was observed in the vessels of the dura and pia mater; and opposite to the part of the skull where he had received the contusion, that is, nearly about the middle of the right parietal bone, there was, in the cortical part of the cerebrum, a blackish spot, about the size of a shilling, which appeared mashed or bruised, and its organization entirely destroyed. There was not the slightest vestige of fissure or fracture in any part of the cranial parietes; no effusion of water in the ventricles, nor a drop of blood effused on any part of the brain. The viscera of the chest and abdomen were perfectly sound.

This Case will shew the difficulty of drawing the line between concussion and compression. Without injury to the skull, there was here as much mischief as if the bone had been shattered, perhaps much more. That the symptoms got worse after venæsection I am ready to allow; but would any plan of treatment have restored vitality to the injured brain in this case? Would any plan of treatment be so likely to restore vitality (provided it were within the reach of cure) as the lessening of the whole volume of blood? But as the injury was beyond the power of art, so the employment of art seemed to accelerate the fatal catastrophe.

JOHN CUNNINGHAM.

*His Majesty's Ship Rochefort,*
*Dec. 5th,* 1817.

